# New onset hidradenitis suppurativa and eruptive milia associated with gamma secretase inhibitor therapy in a patient with desmoid tumor: A case report

**DOI:** 10.1016/j.jdcr.2025.05.042

**Published:** 2025-07-05

**Authors:** Yasmin Gutierrez, Brittney DeClerck, Jennifer L. Hsiao, Katrina H. Lee

**Affiliations:** Department of Dermatology, University of Southern California, Keck School of Medicine, Los Angeles, California

**Keywords:** desmoid tumor, gamma-secretase inhibitor, hidradenitis suppurativa, milia, nirogacestat

## Background

Hidradenitis suppurativa (HS) is a chronic inflammatory disorder that predominantly affects intertriginous sites such as the axillae, inframammary folds, inguinal, and gluteal regions. It is characterized by recurrent nodules, abscesses, and draining tunnels that often lead to irreversible scarring. The etiology of HS is believed to be multifactorial and likely involves a combination of follicular occlusion, genetic factors, hormonal influences, and immune dysregulation.^1^ Recent literature suggests that mutations in the gamma secretase (GS) complex may play a role in driving HS in certain patient subpopulations.^2^ Herein, we present a case of a patient who developed new-onset HS-like nodules, draining abscesses, and eruptive milia following the use of a GS inhibitor.

## Case

A 40-year-old female with a history of anemia, prediabetes, and desmoid fibromatosis presented with a several-month history of draining skin lesions. Four months prior to presentation, she had started treatment with nirogacestat, a GS inhibitor, for desmoid fibromatosis that was previously refractory to sorafenib and pazopanib. Approximately 3 months after initiation of nirogacestat 150 mg twice daily, she began to develop tender bumps on her right axilla and subsequently presented to the emergency department for an expanding abscess requiring incision and drainage. She also noted the development of asymptomatic small bumps on the buttocks, labia, and inguinal folds after starting nirogacestat. The patient denied history of HS or HS-like lesions prior to the initiation of nirogacestat.

**Fig 1:**
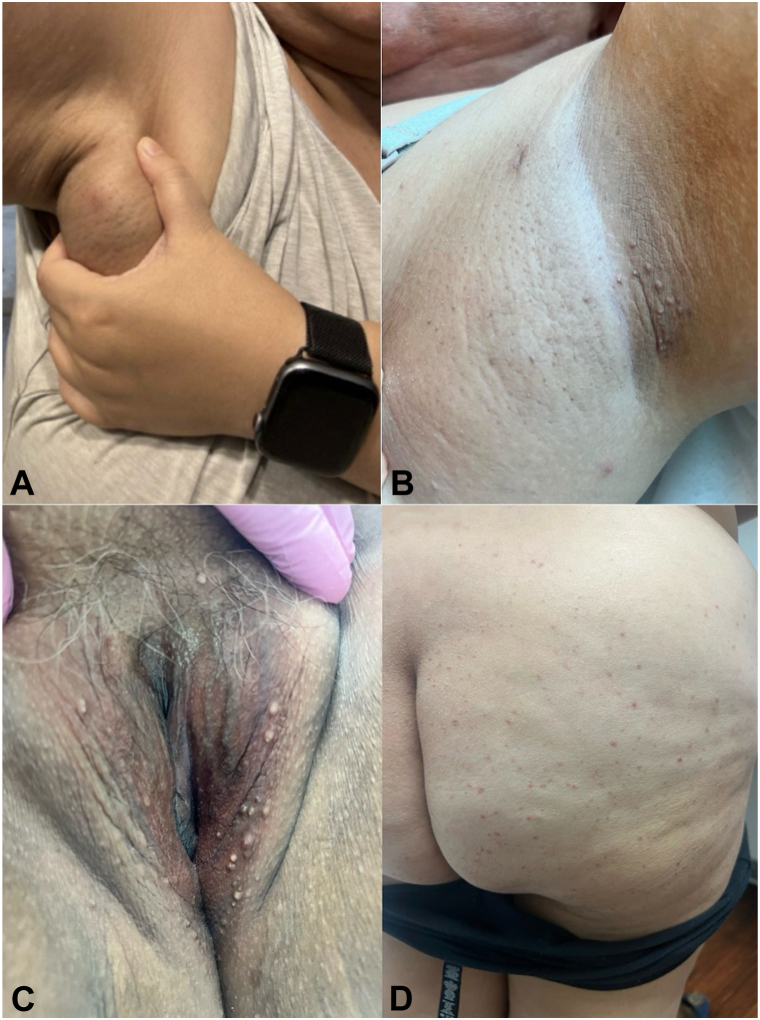
Clinical photos of the patient revealed the following findings. **A,** Subcutaneous fluctuant mass with central erythematous nodule on the right axilla; **(B)** Few follicular papules and scarring on the left axilla; **(C)** Several firm, *white* 1 to 3 mm papules on the labia majora; **(D)** Scattered firm whitish papules, some with erythematous base, on the buttocks.

**Fig 2:**
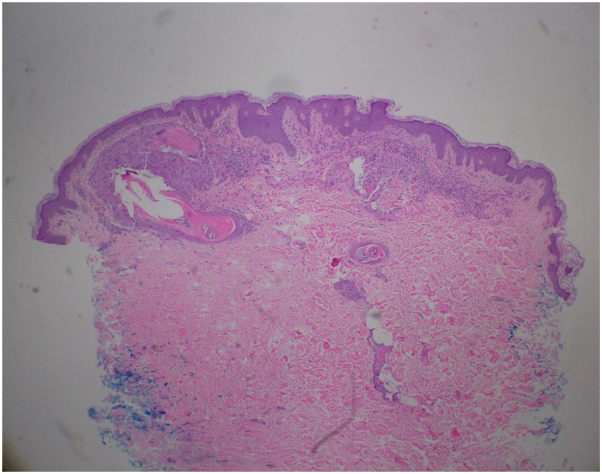
Histopathology at 4× magnification revealed a superficial keratin-filled cavity lined by stratified squamous epithelium consistent with milia.

On examination, the patient was noted to have a healing incision site on the right axilla, an inflamed nodule on the left axilla, scattered firm whitish papules, some with erythematous base, on the buttocks and several small, firm, white 1 to 3 mm papules in the groin, labia, and bilateral axillae (Fig 1). A punch biopsy of one of the white papules revealed features consistent with milia (Fig 2). The patient was diagnosed with HS in her axillae and eruptive milia (some inflamed) on her buttocks and vulva. The patient was started on doxycycline 100 mg twice daily, metformin 500 mg daily, topical clindamycin, and chlorhexidine wash for her HS and topical tretinoin 0.025% cream for the milia.

Two months later, the patient experienced a recurrent flare of the right axillary lesion, which was again treated with incision and drainage. She also noticed the development of new milia lesions on her face. The patient down-titrated her nirogacestat by half and eventually self-discontinued due to her painful and bothersome cutaneous symptoms. Her HS lesions subsided without further flares and her milia resolved within 2 months after discontinuation of nirogacestat.

## Discussion

The exact cause of HS is not yet completely understood, but is thought to involve genetic, environmental, hormonal, and immune influences. The GS complex, an intramembranous protease complex, is composed of 4 proteins (presenilin, presenilin enhancer-2, nicastrin, and anterior pharynx defective 1) and is capable of cleaving more than 30 transmembrane proteins.^3^ Previous literature has suggested that deficiencies of the GS complex may lead to dysfunction in the *Notch* signaling pathway and thus predispose to cyst formation; however, the exact mechanism is still unclear.^4^ Loss-of-function mutations in GS complex have been implicated in a subset of HS patients and are associated with familial HS.^3^

GS inhibitors are a novel class of targeted therapy approved by the US Food and Drug Administration in November of 2023 for progressing desmoid tumors. Early clinical trials of GS inhibitors reported a high incidence of skin toxicity, although the lesions were not fully characterized.^5^ More recent studies, including a phase II trial of nirogacestat in adults with progressive desmoid tumors, reported skin toxicities such as follicular and cystic lesions in 75% of patients.^6^ In a phase III clinical trial involving a similar cohort, hidradenitis-like lesions were observed in 9% of patients, and these lesions were managed with dose modification, topical glucocorticoids, and antibiotics.^7^ However, hidradenitis-like lesions associated with GS inhibitors have not been widely reported in the literature,^6^^,^^8^ likely due to the overall rarity of desmoid tumors and only recently approved targeted therapy.

Notably, our patient also developed eruptive milia, a side effect not commonly associated with GS inhibitors. To our knowledge, there are no reports of eruptive milia occurring after initiation of a GS inhibitor, nor were such events reported in clinical trials. However, the temporal association between the onset of milia and hidradenitis-like lesions in our patient leads us to believe that these cutaneous eruptions were both linked to GS complex inhibition.

GS inhibitors represent a promising therapeutic option for patients with progressing desmoid tumors. Given the known pathogenesis of HS and its association with the GS complex, we anticipate that the increasing use of GS inhibitors will lead to more cases like ours. In our case, the patient discontinued the GS inhibitor due to worsening cutaneous symptoms. It is unclear that the degree to which GS inhibitor induced HS is responsive to traditional HS therapies such as antibiotics, hormonal modulators, metabolic agents, and biologics. However, this case underscores the importance of early recognition of GS inhibitor-induced HS, as timely recognition and management is crucial in minimizing morbidity. If HS symptoms are debilitating for the patient, it may be reasonable to work with the patient’s oncologist to trial dose reduction.

Our case represents one of the few reports of new-onset HS-like lesions induced by GS inhibitors, and to our knowledge, the first to describe the association with eruptive milia. As the use of GS inhibitors becomes more widespread in the treatment of refractory desmoid tumors, it is important for clinicians to be aware of the potential skin toxicities associated with these drugs. Further studies are needed to define the GS complex’s exact role in the pathogenesis of HS and may one day open the door for the development of a new targeted therapeutic approach.

## Conflicts of interest

Dr Hsiao is on the board of directors for the HS Foundation and has served as an advisor, investigator, and/or speaker for AbbVie, Aclaris, Boehringer Ingelheim, Galderma, Incyte, Novartis, Sanofi, Regeneron, and UCB. Dr Lee has served as an investigator for Novartis. The other authors have no other conflicts to disclose.
